# Predictors of hospital admission when presenting with acute-on-chronic breathlessness: Binary logistic regression

**DOI:** 10.1371/journal.pone.0289263

**Published:** 2023-08-15

**Authors:** Ann Hutchinson, Alastair Pickering, Paul Williams, Miriam Johnson

**Affiliations:** 1 University of Hull, Hull, United Kingdom; 2 Emergency Department, Consultant in Emergency Medicine, Hull Royal Infirmary, Hull University Teaching Hospitals Trust, Hull, United Kingdom; San Giuseppe Hospital, ITALY

## Abstract

**Background:**

Breathlessness due to medical conditions commonly causes emergency department presentations and unplanned admissions. Acute-on-chronic breathlessness is a reason for 20% of emergency presentations by ambulance with 69% of these being admitted. The emergency department may be inappropriate for many presenting with acute-on-chronic breathlessness.

**Aim:**

To examine predictors of emergency department departure status in people with acute-on-chronic breathlessness.

**Design, setting and method:**

Secondary analysis of patient-report survey and clinical record data from consecutive eligible attendees by ambulance. Variables associated with emergency department departure status (unifactorial analyses; p<0.05) were included in a binary logistic regression model. The study was conducted in a single tertiary hospital. Consecutive survey participants presenting in May 2015 with capacity were eligible. 1,212/1,345 surveys were completed. 245/1,212 presented with acute-on-chronic breathlessness, 171 of whom consented to clinical record review and were included in this analysis.

**Results:**

In the final model, the odds of admission were increased with every extra year of age [OR 1.041 (95% CI: 1.016 to 1.066)], having talked to a specialist doctor about breathlessness [9.262 (1.066 to 80.491)] and having a known history of a heart condition [4.177 (1.680 to 10.386)]. Odds of admission were decreased with every percentage increase in oxygen saturation [0.826 (0.701 to 0.974)].

**Conclusion:**

Older age, lower oxygen saturation, having talked to a specialist, and having history of a cardiac condition predict hospital admission in people presenting to the emergency department with acute-on-chronic breathlessness. These clinical factors could be assessed in the community and may inform the decision regarding conveyance.

## Introduction

Breathlessness due to medical conditions commonly causes emergency department presentations and unplanned admissions [1, 2]. Its intensity on arrival at the emergency department predicts hospital admission [3]. Acute-on-chronic breathlessness [4] is a reason for one fifth of emergency department presentations by ambulance with between half to two-thirds of these being admitted [1, 5]. The emergency department may be inappropriate for many presenting with acute-on-chronic breathlessness [6], of whom one-third can be discharged home [1, 7]. Avoidable presentations further pressurise an already overstretched service. We explored patient characteristic predictors of emergency department departure status in people with acute-on-chronic breathlessness with the aim of identifying which characteristics might be used by clinicians in the community or in the ED to predict those who would be likely to require admission.

## Materials and methods

We conducted a secondary analysis of survey and clinical record data from those presenting due to acute-on-chronic breathlessness to the major emergencies area of a single tertiary hospital. Primary findings and detailed methods are reported elsewhere [1]. Collected data included: socio-demographic characteristics, medical conditions, breathlessness (severity now/at call-out; duration), respiratory measures on arrival (oxygen saturation, respiratory rate), previous presentations, and the decision-maker regarding emergency call-out (self/carer/clinician).

Patient characteristic candidate variables regarding association with emergency department departure status were those i) with a plausible biological explanation, ii) from the published literature, iii) identified by unifactorial analyses (p<0.05).

Independent samples Z-tests were performed for all continuous predictor variables, Pearson Chi squared test for unordered categorical data with more than two groups, Fisher’s Exact test for binary categorical variables and Kendall’s Tau-b when dependent variables were ordinal with more than two levels.

Candidate variables were included in a binary logistic regression using stepwise analysis with backwards elimination to predict emergency department departure status. In this preliminary analysis, we applied the recommended rule of at least 10 positive cases and 10 negative cases [8] *per* predictor variable. Given our sample size of 171 complete cases (120 admitted and 51 discharged) the acceptable number of independent variables to be entered into a binary logistic regression was ≤ five. Therefore, only the five most strongly associated variables from the unifactorial analysis were used in the final model. With similarly strongly associated variables likely to have collinearity (e.g. self-report or case record diagnosis of heart disease), the variable with the highest odds ratio/best biological rationale was chosen. Analysis was undertaken using SPSS (Released 2011. IBM SPSS Statistics for Windows, Version 20.0).

For the primary data collection, ethics approval, including for the method of consent, was given by NHS National Research Ethics Service Committee South Central-Hampshire B (Ref: /SC/0543). Implied consent was given for participation by completion of the survey, however, consent did not extend for the use of their data by researchers outside this research team. For this secondary analysis, no further permissions were required.

## Results and discussion

### Sample data

During the survey period (12/5/2014 to 29/5/2014), 1,345/2,041 emergency department attendees were eligible; 1,212/1,345 surveys were completed. Breathlessness most days over the past month was self-reported by 424/1,212 and acute-on-chronic breathlessness was a reason for presentation in 245/424; 177/245 consented to clinical record review and data were extracted between 30/5/2014 and 31/7/2014. 171 complete cases were analysed.

The 245 with acute-on-chronic breathlessness had an average age of 65 (+/-19) years, 117/245 were men and most described moderate (72/236 mMRC grade 3) or severe (87/236 mMRC grade 4) breathlessness over the previous month. About half (112/237) had breathlessness for over 2 years.

### Selection of potential variables associated with emergency department departure status: Unifactorial analysis

Forty-eight independent clinical and demographic variables (extracted from both the survey and clinical record) were assessed for significant (p<0.05) univariate association with emergency department departure status (data available on request). Seven candidate patient characteristic predictors were identified as being very strongly significantly associated with emergency department departure status (see Table 1). Three related to a diagnosis of heart disease. In view of the likely collinearity “Case-record history of heart disease” was chosen because of little difference in statistical significance, a larger odds ratio, and biological plausibility to include ischaemic heart and other diseases as well as heart failure.

**Table 1:** Predictor variables associated with admission to hospital in patients presenting to the emergency department due to breathlessness.

Candidate predictor variables*	Coefficient	95% confidence intervals	Unifactorial analysis (P value)	Final Regression model (OR, 95% CI and P value)
Age	Mean difference = 18yrs	12–24	<0.001	1.041 per year of age (1.016–1.066) **P = 0.001**
Oxygen saturation	Mean difference = -1.669	-2.587 to -0.750	<0.001	0.826 per every % point increase (0.701–0.974) **P = 0.023**
Talk to specialist doctor	OR = 12.00	1.57 to 91.48	0.002	9.262 (1.066–80.491) **P = 0.044**
Self-reported diagnosis—any heart condition	OR = 2.65	1.34 to 5.24	0.005	NA
Case-record history—any heart condition**	OR = 4.69	2.21 to 10.00	0.001	4.177 (1.680–10.386) **P = 0.002**
Case-record history of heart failure	OR = 4.27	1.78 to 10.20	<0.001	NA
Charlson comorbidity	Tau-b = 0.267		<0.001	Dropped after step one elimination

* Only the variables most strongly associated with admission in the univariable analysis are shown.

** Only this heart disease variable included in the stepwise regression in the final model because greater odds and better biological rationale

### Predictors of emergency department departure status: Multifactorial analysis

The five most strongly correlated included variables were: age, oxygen saturation at presentation, having talked to a specialist doctor about breathlessness, a documented heart condition and the Charlson comorbidity score.

In the final model, the odds of admission were increased with every extra year of age [OR 1.041 (95% CI: 1.016 to 1.066)], having talked to a specialist doctor about breathlessness [9.262 (1.066 to 10.386)] and a known heart condition [4.177 (1.680 to 10.386)] and were decreased with every percentage increase in oxygen saturation [0.826 (0.701 to 0.974)] (see Table 1).

### Summary

Our exploratory study showed the odds of hospital admission resulting from emergency department presentation due to acute-on-chronic breathlessness increased with increasing age, decreasing oxygen saturation, having talked to a specialist doctor about breathlessness and having a heart condition.

### Strengths and limitations

Our consecutive survey sample, a 90% response rate and minimal missing data are strengths, although seasonal variation was not addressed. However, of the 245 presentations due to acute-on-chronic breathlessness, only 177 consented to clinical record review with 171 complete records obtained introducing selection bias. The study was conducted in one site, but produced findings similar to a multi-site study [7]. These findings are not representative of all patients presenting to the emergency department with acute-on-chronic breathlessness; especially those with very severe breathlessness requiring immediate resuscitation, who lacked capacity to complete the survey.

Our study is exploratory, was not designed to develop a prediction model, and data were collected *post* conveyance to the ED. As we could only investigate a few strongly associated variables, other important ones may have been missed. We did not include variables relating to treatment or laboratory investigations. However, the tested variables can be assessed in the community and are therefore relevant for conveyance triage. The data were collected six years ago however, the predictors identified remain relevant.

### Comparison with existing literature

Around two-thirds of people attending the emergency department with breathlessness are admitted to hospital [1, 7, 9–11]. A disproportionately high admission rate of 86% was also noted in the heart failure substudy of the AANZDEM cohort [12]. Half of our sample were admitted for only one day. Collins and colleagues [13] also noted that over half of those admitted with heart disease from the emergency department were discharged within a brief period. The high proportion of admissions for people with heart disease, compared with lung disease, probably reflects the greater need for hospital-based investigations and intensive medical management.

A number of prediction models exist regarding admission from the emergency department. Older age is a consistently important variable [14–17], consistent with increased likelihood of multi-morbidity and frailty, coupled with lack of social support and greater care needs. The chief complaint is also a recognised predictor, and older adults are more likely to present to the emergency department with breathlessness [18]. The importance of oxygen saturation in triage is well recognised [19], although pulse oximetry may miss some who require admission [20].

Tachycardia is associated with admission in people with chronic heart failure and chronic obstructive pulmonary disease [5, 10, 14], although less helpful in older adults [18], but we did not record this variable. Arrival by ambulance to the emergency department, tachycardia and very severe self-reported breathlessness (numerical rating scale severity of ≥8/10) predicted hospital admission, as did increased distress due to breathlessness [3]. With regard to breathlessness severity, Saracino et al. [3] assessed this on entry to the emergency department, whilst we assessed patients sufficiently stable to be offered the survey, and their breathlessness had settled whilst waiting.

Self-management of acute-on-chronic breathlessness using cognitive [21] and other non-pharmacological [22] interventions reduces hospital admission, and some patients become expert [23, 24]. Interventions to foster a feeling of safety at home and control of breathlessness may reduce presentations to the emergency department; sometimes the mere arrival of a paramedic reduces anxiety-driven acute-on-chronic breathlessness [6]. This is the premise of a current feasibility trial of a paramedic-delivered non-pharmacological intervention (ISRCTN number 80330546) [25].

### Implications for research and/or practice

The identified predictors are simple and non-invasive, measurable by community clinicians. Our findings may be useful to aid clinical decision-making regarding conveyance to the emergency department and help prevent attendance by those who settle quickly and would be discharged back home. Additionally, these predictors might be useful at triage in the emergency department to expedite the admission of those most in need. Confirmation of our findings with a larger dataset from community-collected data is needed before being applied in clinical practice. Designs could include quasi-experimental approaches based on propensity score matching to address confounding to test different possible interventions and the usefulness of candidate predictors in clinical practice.

## Conclusions

Admission from the emergency department was predicted by increased age, decreased oxygen saturation on presentation, having talked to a specialist doctor about breathlessness, and a history of a cardiac condition. These clinical factors can be measured by community clinicians and consideration may result in fewer unnecessary emergency department presentations with more patients being managed appropriately in the community.
